# Age Limits of Breast Cancer Screening with Mammography—A Decision-Analytic Benefit–Harm Evaluation to Inform Decision Making for the German Context

**DOI:** 10.3390/cancers18172750

**Published:** 2026-08-25

**Authors:** Gaby Sroczynski, Lára R. Hallsson, Nikolai Mühlberger, Felicitas Kühne, Beate Jahn, Christin Henning, Heike Kölsch, Stefan Sauerland, Konstanze Angelescu, Uwe Siebert

**Affiliations:** 1 Institute of Public Health, Medical Decision Making and Health Technology Assessment, Department of Public Health, Health Services Research, and Health Technology Assessment, UMIT TIROL—University for Health Sciences and Technology, 6060 Hall in Tirol, Austria; 2 Division of Health Technology Assessment, ONCOTYROL—Center for Personalized Cancer Medicine, 6020 Innsbruck, Austria; 3 Institute for Quality and Efficiency in Health Care (IQWiG), 50679 Cologne, Germany; 4 Center for Health Decision Science, Department of Health Policy & Management, Harvard T. H. Chan School of Public Health, Boston, MA 02115, USA; 5 Department of Epidemiology, Harvard T. H. Chan School of Public Health, Boston, MA 02115, USA; 6 Department of Radiology, Massachusetts General Hospital, Harvard Medical School, Boston, MA 02115, USA; 7 Center for Health Technology Assessment, Mass General Brigham, Harvard Medical School, Boston, MA 02145, USA

**Keywords:** benefit–harm relation, breast cancer screening, mammography, decision analysis

## Abstract

**Background/Objectives**: To inform policy making for the German breast cancer (BC) screening program, we systematically evaluated the long-term benefits and harms of extended age limits compared to the current standard of biennial mammography at ages 50–69 years using a decision-analytic approach. **Methods**: We developed and applied a Markov state-transition model for mammography screening in Germany to systematically assess the benefit–harm trade-offs of various screening strategies varying in age at start and end of screening as well as in screening frequency. The model was populated with international data for sensitivity and specificity of mammography along with German epidemiological, clinical and age-specific quality-of-life data. In deterministic analyses, the following outcomes were projected: detected ductal carcinoma in situ (DCIS) and invasive BC, BC-related deaths, life years (LY), and quality-adjusted life years (QALY), number of positive, false-positive, and total mammograms, overdiagnosis, and the incremental harm–benefit ratio (IHBR). **Results**: In the base-case analysis, mammography at ages 45–79 (annual, age 45–49; biennial, 50–79) achieved the highest gain in LY (10.0 life years gained [LYG] per 100 women) compared with current screening. Biennial mammography at ages 45–74 resulted in the highest benefits considering both life expectancy and quality of life (3.5 QALYs gained/100 women). Compared to current biennial mammography screening at ages 50–69, lowering the start age from 50 to 45 years resulted in an IHBR of 47 additional mammograms/LYG. Compared to biennial mammography at ages 45–69, biennial mammography at age 45–74 results in 96 additional mammograms/LYG. Further extended screening results in substantially less favorable IHBRs. **Conclusions**: Based on our results, extending biennial mammography screening to women aged 45 to 74 years may prevent additional BC deaths and increase remaining life expectancy at an acceptable benefit–harm ratio, and improve quality-adjusted life expectancy.

## Simple Summary

Breast cancer screening in Germany currently invites women aged 50–69 for a mammogram every two years. We used a Markov Model considering German data and practice patterns to compare several screening strategies that start at younger ages, end later, and varied how often mammograms are performed. We looked at patient-relevant outcomes such as breast cancer cases and deaths prevented, life expectancy, quality of life, and the number of extra mammograms and false-positives—to weigh benefits against burdens. Our results suggest that inviting women aged 45–74 for a mammogram every two years could prevent more breast cancer deaths and modestly extend both life expectancy and quality-adjusted life expectancy with an acceptable trade-off in additional testing, while more extensive screening brings less favorable incremental harm–benefit ratios. For policymakers, the results offer country-specific transparent evidence to refine screening guidelines.

## 1. Introduction

The World Health Organization reported for the year 2022 about 2.3 million new breast cancer cases and 670,000 breast cancer deaths, worldwide [1]. In Germany, breast cancer is the most prevalent cancer among women, with about 74,500 newly diagnosed cases and an age-standardized incidence of 117.5 per 100,000 persons (European population standard) in the year 2022 [2]. The estimated lifetime risk of women in Germany for developing breast cancer is 13.2% (one in eight women develop breast cancer in their lifetime) with a lifetime risk of dying from breast cancer of 3.5%. Due to changes in lifestyle, aging population and other increasing risk factors associated with breast cancer development, lifetime risk for breast cancer is projected to further increase within the coming decades [3].

In 2005 Germany launched its organized breast cancer screening program with biennial mammography for women at ages 50 to 69 years, that may have the potential to reduce cancer mortality [4]. Recent European guidelines for breast cancer screening recommend extending the organized mammography screening for all women to age 45–74 years [5]. On this basis, the German Federal Joint Committee (G-BA), the paramount decision-making body within the German health care system, commissioned the Institute for Quality and Efficiency in Health Care (IQWiG) to review the age limits of the German mammography screening program. As part of this benefit assessment based on empirical studies, a decision-analytic modeling study was conducted to systematically assess the benefit–harm trade-offs for different mammography screening strategies that vary by the lower and upper age to begin and end screening, and corresponding screening intervals. While results of decision-analytic modeling studies are increasingly used internationally in the development of guidelines (e.g., US Preventive Services Task Force (USPSTF)) and to support policy decisions [6,7], this modeling study was the first application of its kind within a benefit assessment of the IQWiG in the German health care context.

Here, we report on the model-based long-term benefits and harms of different breast cancer screening strategies with mammography that vary by lower and upper age limits, and screening intervals. Screening strategies included current practice in Germany, and were extended according to the most recent scientific findings from European and international guidelines [5,6,7,8]. The results aim to complement empirical findings from the benefit assessment conducted by IQWiG [8] to support policy recommendations.

## 2. Materials and Methods

### 2.1. Model Design and Screening Strategies

**Figure 1:**
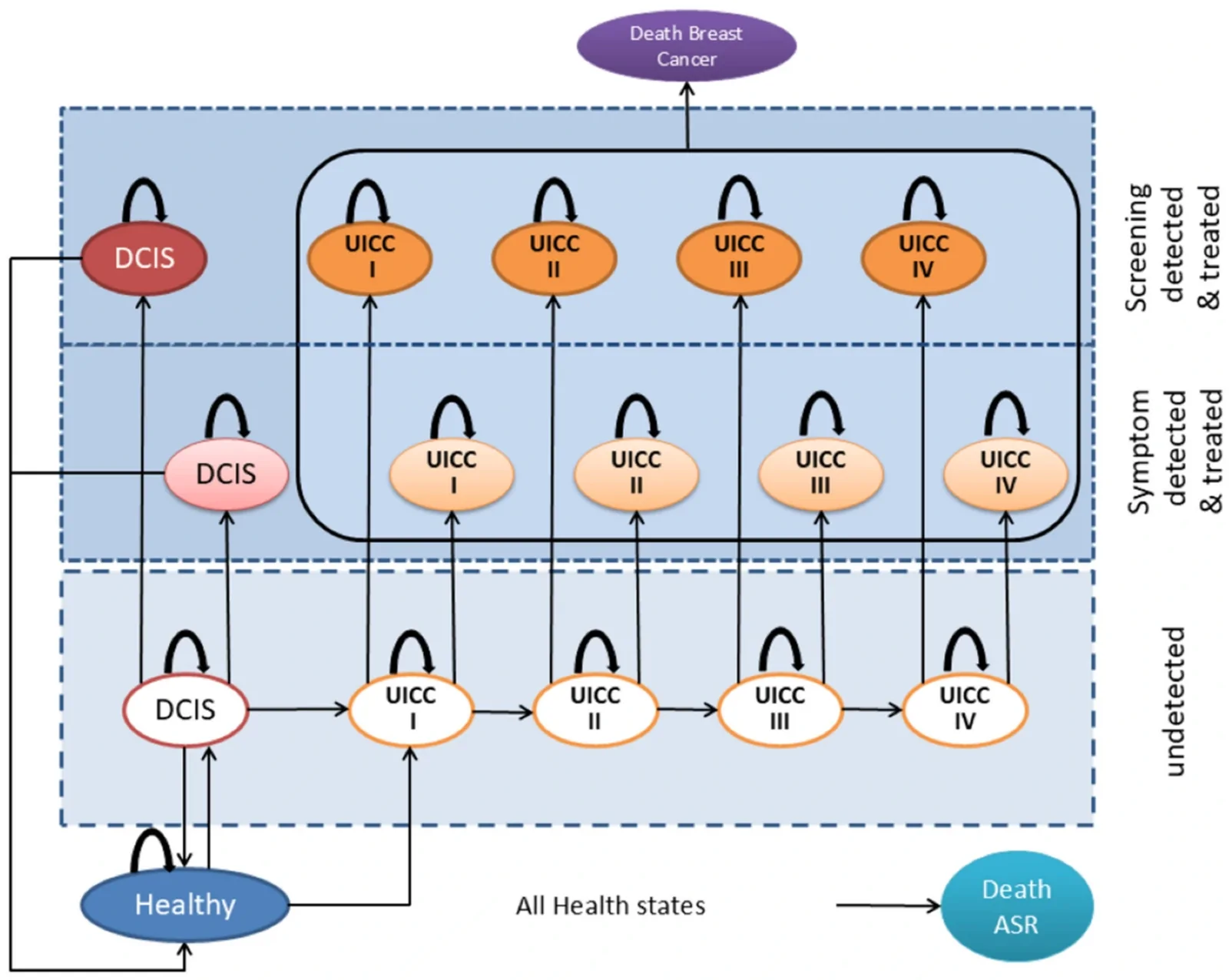
Model schematic: natural history of breast cancer development and detection in the Markov state-transition model (based on [8]). ASR—age, sex, race, DCIS—ductal carcinoma in situ, UICC—Union for International Cancer Control classification. Each bubble represents a health state. Each arrow represents possible transitions between health states that occur each year with specific transition probabilities.

We developed, calibrated and validated a Markov state-transition model simulating breast cancer development (Figure 1) including ductal carcinoma in situ (DCIS), screening, detection, and management for women in Germany. The model design follows the international ISPOR-SMDM Modeling Good Research Practices for decision-analytic modeling and key principles for health technology assessment [9,10,11].

In this model, a hypothetical cohort of initially healthy women moves in annual cycles through different health states over their lifetime. Women may develop DCIS and progress to invasive breast cancer or may directly develop invasive breast cancer. DCIS may progress to invasive breast cancer or regress. Preclinical (i.e., undetected) carcinoma can progress from stage I to stage IV based on the Union for International Cancer Control (UICC) classification. Both DCIS and invasive carcinomas can be detected through screening and symptoms presentation and are treated according to German clinical guidelines [12]. Women with an initial cancer diagnosis may die from invasive breast cancer, based on stage-specific mortality rates, or from other causes, or remain in the disease state for the remainder of their lives. At any point in time, women may die from causes other than breast cancer based on the age- and sex-specific mortality probabilities. Regular and complete participation in screening and follow-up treatments are adopted.

All women start in a healthy state. Over time, women may develop undetected DCIS or undetected stage UICC I breast cancer. Undetected DCIS may regress to the healthy state. Undetected stage UICC I breast cancer can progress to advanced stages UICC II-IV. Undetected DCIS and undetected cancer can be diagnosed at any stage by symptoms or screening. Women with diagnosed DCIS or diagnosed cancer (through symptoms or screening) move on to their respective diagnosed health condition and receive treatment appropriate to the diagnosis. Women diagnosed with DCIS may progress to a condition with undetected UICC I stage breast cancer. Women diagnosed with breast cancer may die from breast cancer, with the probability of death, and therefore the time to death, being determined by the initial tumor stage detected. In all health states women may die due to other causes according to the age- and sex-specific mortality in Germany.

The model compares 14 different strategies differing by age at start (45 or 50 years) and end of screening (65, 69, 74 or 79 years), as well as by screening interval (annual, biennial or triennial) using the current screening standard in Germany as the reference strategy (Table 1).

**Table 1:** Screening strategies evaluated.

No.	Screening Strategy: Screening Test, Interval, Age at Start and End
1.	No screening
2.	Mammography, biennial, age 50–69 years (reference strategy ^1^)
3.	Mammography, annual, age 45–49 years; biennial, age 50–69 years
4.	Mammography, biennial, age 45–69 years
5.	Mammography, annual, age 45–49 years; biennial, age 50–74 years
6.	Mammography, biennial, age 45–74 years
7.	Mammography, annual, age 45–49 years; biennial, age 50–79 years
8.	Mammography, biennial, age 45–79 years
9.	Mammography, annual, age 45–49 years; biennial, age 50–69 years; triennial, age 70–74 years
10.	Mammography, annual, age 45–49 years; biennial, age 50–69 years; triennial, age 70–79 years
11.	Mammography, biennial, age 50–74 years
12.	Mammography, biennial, age 50–79 years
13.	Mammography, biennial, age 50–69 years; triennial, age 70–74 years
14.	Mammography, biennial, age 50–69 years; triennial, age 70–79 years

^1^ Current screening standard in Germany.

### 2.2. Model Parametrization, Calibration and Validation

We used German demographic and epidemiological data derived from the German Statistical Agency [13] and the German Cancer Registry [14,15] for model calibration (Table 2). Initial parameter values were based on published literature [16] and adjusted to reflect observed age-specific DCIS, invasive breast cancer incidences and the detected cancer stage distribution in Germany prior to the introduction of breast cancer screening. DCIS and invasive breast cancer development were modeled as a function of age and breast density in the German female population [17,18,19]. Age-specific probabilities to die from causes other than invasive breast cancer were modeled using age-specific other-cause mortality, based on reported age-specific all-cause mortality of the German female population [20] adjusted for breast cancer mortality by age [14]. Breast cancer-specific mortality was based on stage-specific relative survival rates derived from a German cancer registry [21]. We considered improved prognosis for screen-detected versus symptom-detected breast cancer cases using a relative risk of 1.36 of dying from breast cancer for symptom-detected cases based on data from three large breast cancer screening studies [22].

**Table 2:** Model parameters of the natural history of breast cancer.

Transition From	to	Age (Years)	Annual Transition Probability	Source
Healthy	DCIS undetected	0–12	0	calibrated ^1^
		13–29	0.000068	
		30–39	0.002047	
		40–45	0.004536	
		45–49	0.004353	
		50–54	0.004288	
		55–59	0.003184	
		60–64	0.002534	
		65+	0.000122	
Healthy	UICC I undetected	0–14	0	calibrated ^2^
		15–19	0.000001	
		20–24	0.000018	
		25–29	0.000184	
		30–34	0.000449	
		35–39	0.000663	
		40–44	0.001402	
		45–49	0.001611	
		50–54	0.002361	
		55–59	0.002802	
		60–64	0.002910	
		65+	0.002993–0.003311	
DCIS undetected	Healthy		0.068184	Schiller-Fruehwirth 2017 ([16]), calibrated ^2^
	DCIS symptom detected ^2^		0.009446	
	UICC I undetected		0.123799	
UICC I undetected	UICC I symptom detected ^2^		0.163963	
	UICC II undetected ^2^		0.339696	
UICC II undetected	UICC II symptom detected ^2^		0.394884	
	UICC III undetected ^2^		0.391166	
UICC III undetected	UICC III symptom detected ^2^		0.528699	
	UICC IV undetected ^2^		0.619020	
UICC IV undetected	UICC IV symptom detected ^2^		0.829060	

UICC—Union for International Cancer Control classification, DCIS—ductal carcinoma in situ. ^1^ Calibrated to age-specific DCIS incidence for women in Germany before introduction of screening (year 2003) [13]. ^2^ Calibrated to age-specific incidence and UICC stage distribution of detected breast cancer cases before introduction of screening [14].

Evidence on mammography performance as a function of breast density, age and screening interval was derived from published international literature (Table 3) [19,23]. German quality-of-life data (EQ-5D) as a function of age and gender in the general population [24] were multiplied with relative utility values (EQ-5D) for DCIS and breast cancer by stage and age from a Swedish study [25,26] as a function of age to account for the disease-related quality of life. Absolute utility decrements for being screened [17] and experiencing positive findings [27] for a specific time period were applied to account for a temporary decline in quality of life (Table 4).

**Table 3:** Test accuracy of mammography as a function of breast density category, age, and screening interval [19].

Breast Density	Age (Years)	Screening Interval ^1^	Sensitivity (Invasive Cancer)	Sensitivity (DCIS)	Specificity
ACR A	40–49	initial	0.921	0.954	0.872
		annual	0.806	0.919	0.930
	50–64	biennial	0.881	0.942	0.925
		initial	0.948	0.955	0.903
		annual	0.868	0.921	0.948
		biennial	0.921	0.943	0.944
	≥65	initial	0.963	0.955	0.916
		annual	0.903	0.922	0.955
		biennial	0.943	0.944	0.952
ACR B	40–49	initial	0.894	0.948	0.797
		annual	0.751	0.911	0.884
		biennial	0.844	0.935	0.876
	50–64	initial	0.930	0.949	0.843
		annual	0.826	0.912	0.912
		biennial	0.895	0.937	0.906
	≥65	initial	0.950	0.950	0.863
		annual	0.871	0.913	0.924
		biennial	0.924	0.937	0.919
ACR C	40–49	initial	0.817	0.964	0.760
		annual	0.615	0.937	0.860
		biennial	0.740	0.955	0.851
	50–64	initial	0.876	0.965	0.812
		annual	0.716	0.938	0.894
		biennial	0.818	0.956	0.886
	≥65	initial	0.909	0.965	0.836
		annual	0.782	0.938	0.908
		biennial	0.865	0.956	0.901
ACR D	40–49	initial	0.746	0.943	0.815
		annual	0.512	0.902	0.895
		biennial	0.652	0.929	0.888
	50–64	initial	0.822	0.944	0.857
		annual	0.623	0.904	0.921
		biennial	0.747	0.930	0.915
	≥65	initial	0.868	0.944	0.876
		annual	0.702	0.904	0.932
		biennial	0.808	0.931	0.927

ACR BI-RADS—American College of Radiology Breast Imaging-Reporting and Data System, DCIS—ductal carcinoma in situ; ACR A: almost completely fatty (<25% glandular); ACR B: scattered fibro glandular densities (25–50% glandular); ACR C: irregularly dense (51–75% glandular); ACR D: extremely dense (>75% glandular). ^1^ cancer detected by screening diagnosed in one year: cut-off at next screening.

**Table 4:** Health-related quality of life: absolute and relative utility values.

**Quality of Life German Female Population (EQ-5D)**				
	**Age (Years)**		**Utility**	**Source**
	0–17		1	[24]
	18–24		0.950	
	25–34		0.949	
	35–44		0.943	
	45–54		0.908	
	55–64		0.881	
	65–74		0.838	
	75+		0.771	
**Age- and stage-specific relative utility for DCIS and breast cancer ^1,2^**				
**State**	**Age (years)**	**Duration**	**Relative utility**	**Source**
DCIS	0–39	First year	1	Calculated based on Swedish data [17,25,26]
		Further years	1	
	40–49	First year	0.905	
		Further years	1	
	50–59	First year	0.889	
		Further years	1	
	60–80+	First year	0.904	
		Further years	1	
UICC I	0–39	First year	1	
		Further years	1	
	40–69	First year	0.846	
		Further years	0.980	
	70–80+	First year	0.847	
		Further years	0.980	
UICC II	0–39	First year	1	
		Further years	1	
	40–79	First year	0.753	
		Further years	0.905	
	80+	First year	0.753	
		Further years	0.906	
UICC III	0–39	First year	1	
		Further years	1	
	40–79	First year	0.753	
		Further years	0.905	
	80+	First year	0.753	
		Further years	0.906	
UICC IV	0–39	First year	1	
		Further years	1	
	40–59	First year	0.753	
		Further years	0.832	
	60–79	First year	0.753	
		Further years	0.833	
	80+	First year	0.753	
		Further years	0.832	
**Utility decrement for screening (TTO) ^3^**				
	**Absolute** **utility decrement**	**Duration**	**Absolute utility** **decrement over one year**	**Source**
Mammography	0.196	2 weeks	0.0075	[17]
Positive test result	0.105	5 weeks	0.0100	[27]

DCIS: ductal carcinoma in situ; EQ-5D—European Quality of Life 5 Dimensions; UICC—Union for International Cancer Control classification; TTO—time trade-off. ^1^ Age-specific utility values based on the EQ-5D quality-of-life instrument for the German female population were weighted by disease-specific relative EQ-5D utility values as a function of age. German age-specific quality of life was based on a publication by Janssen et al. [24] and multiplied by age-specific relative utility values for DCIS and breast cancer from a Swedish study [17,25,26]. ^2^ Relative utility depends on age and year after diagnosis. ^3^ Absolute utility decrements for mammography [17] and for a positive test result [27] were considered based on the time trade-off (TTO) method.

Further details on model calibration procedure and input parameters are reported in the supplemental material (Supplementary Materials, Tables S1–S3). The model was validated on several levels including face validity, internal validation, and external validation with observed German epidemiological data after breast cancer screening implementation from the cancer registry and published literature [28,29].

### 2.3. Analyses and Outcomes

We performed deterministic cohort simulations evaluating clinical and economic outcomes over a lifelong time horizon. Outcomes consist of the lifetime risks (in %) for detected invasive breast cancer, detected DCIS, and for dying from detected invasive breast cancer; remaining life expectancy (in life years (LY)) per 100 women; remaining quality-of-life-adjusted life expectancy (in quality-adjusted life years (QALY)) per 100 women; absolute number of screening findings (mammograms) per 100 women; absolute number of positive and false-positive screening findings (mammograms) per 100 women; lifetime risk of overdiagnosis (DCIS and breast cancer) (in %) and number needed to screen (NNS) to avoid one breast cancer-related death.

Overdiagnosis refers to the diagnosis of a disease that would never have been detected or would never have caused symptoms without screening. Overdiagnosis does not improve health, but it can cause stress, unnecessary worry, and a decline in quality of life. In this decision-analytic study, a diagnosis of DCIS or invasive cancer detected by mammography was classified as an overdiagnosis in case it would not have been diagnosed at any point during a woman’s lifetime under the “no screening” strategy.

QALY is a generic measure used to quantify the health-related benefits of a health technology (e.g., screening), in which the benefits, in terms of length of life, are adjusted to reflect the quality of life. QALYs were calculated by weighting the time women spent in a given health state by the respective utility values for health-related quality of life in that state. A weight of 1 represents full health-related quality of life, and a weight of 0 is equivalent to death. The cumulative weighted years of life over the lifetime time horizon reflect the quality-of-life-adjusted remaining life expectancy.

In addition to this QALY approach to quantify the integrated benefits and harms, we used an incremental harm–benefit approach to assess and visualize the trade-off between benefits and harms. The incremental harm–benefit ratio (IHBR) is defined as the difference in harms (e.g., psychological and physical harm associated with additional mammograms) between two interventions divided by the respective difference in benefits (e.g., life years gained (LYG)). This ratio expresses the number of harms one must ‘accept’ for ‘gaining’ one additional unit of benefit (e.g., additional mammograms per life year gained).

Currently, there is no empirical evidence on such acceptability threshold or its range or distribution for the German mammography screening context. Importantly, as this threshold for choosing a specific strategy may vary across women, the decision for adopting a specific approach should ultimately be based on the preferences and values of the individual woman.

To evaluate the robustness of the base-case findings and identify influential model parameters, deterministic one-way and multi-way sensitivity analyses were conducted. The analyses explored the impact of varying sensitivity and specificity of mammography as well as quality-of-life data. Multi-way sensitivity analyses were performed to examine the effect of simultaneously varying the sensitivity and specificity of mammography in opposite directions by an absolute ± 5% each on benefits measured by remaining life expectancy (in LYs per 100 women) and quality-of-life-adjusted life expectancy (in QALYs per 100 women).

TreeAge Pro Healthcare software (version 2021, R2.0) [30] was used for developing and analyzing the decision-analytic model. Microsoft Excel [31] was used for model parameter transformations.

## 3. Results

### 3.1. Model Calibration and Validation

Model calibration results show a good fit between the modeled data and the observed data for the age-specific annual incidence rate of symptomatically detected DCIS (Supplementary Materials, Figure S1), the age-specific annual incidence rate of symptomatically detected breast cancer (Figure S2) and the UICC stage distribution of detected breast cancer cases (Figure S3). With an average screening participation of 50% in the biennial screening program with mammography for women at ages 50–69, the model predicted a lifetime risk of 11.5% for being detected with invasive breast cancer and 3.5% to die from breast cancer. In Germany, for the year 2019 the reported lifetime risks for detected breast cancer and death from breast cancer were 13.2% and 3.5%, respectively [32]. In addition, the model estimated over a lifetime a distribution of 82.4% for early-stage cancer (DCIS + UICC I) and 17.6% for stages UICC II-IV in women screened biennially at ages 50 to 69. The annual report of the German breast cancer screening program reported 78.6% for early stages (DCIS + UICCI) and 21.4% for stages UICC II-IV in women who have been screened in the year 2018 excluding initially screened women [29].

### 3.2. Absolute Benefits and Harms

Table 5 shows the average remaining life years and QALYs for each screening strategy for a cohort of 100 women at age 45 years. On average, mammography compared to no screening increases the remaining life expectancy by an average of 22 to 32 life years (12.5 to 16 QALYs) per 100 women, depending on intensity and duration of screening (i.e., screening interval and age at start and end of screening). The greatest gain in life years is achieved with the most intensive mammography screening at ages 45 to 79 years with an annual interval for women younger than 50 and biennial screening for women 50 years and older. Considering quality of life, biennial mammography screening at ages 45 to 74 achieves the highest incremental benefit (3.5 QALYs gained/100 women vs. current screening). Lowering the age for screening initiation yields a greater incremental benefit in terms of life years and QALYs gained than increasing the upper age limit.

**Table 5:** Base-case analysis: remaining life expectancy and quality-of-life-adjusted residual life expectancy per 100 women.

	Remaining Life Expectancy per 100 Women (After the Age of 45 Years)			Quality-of-Life-Adjusted Remaining Life Expectancy per 100 Women (After the Age of 45 Years)		
Strategy	Life Years	Incremental Life Years vs. No Screening	Incremental Life Years vs. Reference Strategy	QALYs	Incremental QALYs vs. No Screening	Incremental QALYs vs. Reference Strategy
No screening	3847.7	-	-	3310.0	-	-
Screening: age 50–69 y, 2 y (Ref)	3870.0	22.4	-	3322.5	12.5	-
Screening: age 50–69 y, 2 y; 70–74 y, 3 y	3871.2	23.5	1.1	3322.9	13.0	0.5
Screening: age 50–74 y, 2 y	3872.5	24.8	2.4	3323.1	13.1	0.6
Screening: age 50–69 y, 2 y; 70–79 y, 3 y	3872.6	25.0	2.6	3323.2	13.3	0.8
Screening: age 50–79 y, 2 y	3873.4	25.7	3.3	3322.9	13.0	0.4
Screening: age 45–69 y, 2 y	3876.0	28.4	6.0	3325.6	15.6	3.1
Screening: age 45–49 y, 1 y; 50–69 y, 2 y	3877.1	29.4	7.0	3325.0	15.0	2.5
**Screening: age 45–74 y, 2 y**	3877.7	30.0	7.6	**3326.0**	**16.0**	**3.5**
Screening: age 45–49 y, 1 y; 50–69 y, 2 y; 70–74 y, 3 y	3878.1	30.4	8.1	3325.4	15.4	2.9
Screening: age 45–49 y, 1 y; 50–74 y, 2 y	3878.7	31.0	8.6	3325.4	15.4	2.9
Screening: age 45–79 y, 2 y	3879.0	31.4	9.0	3325.8	15.8	3.3
Screening: age 45–49 y, 1 y; 50–69 y, 2 y; 70–79 y, 3 y	3879.4	31.7	9.3	3325.6	15.6	3.1
**Screening: age 45–49 y, 1 y; 50–79 y, 2 y**	**3880.0**	**32.4**	**10.0**	3325.2	15.2	2.7

y—years; QALYs—quality-adjusted life years, Ref—reference strategy. Bold: the strategies with highest incremental benefit in terms of remaining life expectancy and quality-adjusted remaining life expectancy.

Table 6 shows the average health-related event frequencies regarding various benefit and harm endpoints expected for the different screening strategies evaluated. Both benefits and potential harms increase with increasing intensity and duration of mammography screening.

**Table 6:** Base-case analysis: predicted event frequencies of various benefit and harm endpoints for each screening strategy (based on [8]).

Strategy	Lifetime Risk: Detected BC	Lifetime Risk: Detected DCIS	Lifetime Risk: BC Death	Mammograms per 100 Women	Positive Mammograms per 100 Women	False-Positive Mammograms per 100 Women	Lifetime Risk: Over-Diagnosis (DCIS + BC)	NNS to Prevent One BC-Related Death
	(%)	(%)	(%)	(n)	(n)	(n)	(%)	(n)
No screening	11.12	1.01	4.27	0	0	0	0	-
Screening: age 50–69 y, 2 y (Ref)	10.78	7.65	3.05	903	98.4	86.7	6.30	81
Screening: age 50–74 y, 2 y	10.83	7.71	2.80	1135	117.7	104.8	6.41	68
Screening: age 50–79 y, 2 y	10.89	8.67	2.68	1273	129.2	115.4	6.55	63
Screening: age 45–69 y, 2 y	10.70	7.76	2.80	1186	136.5	123.1	7.18	68
Screening: age 45–74 y, 2 y	10.74	8.70	2.64	1341	149.4	135.1	7.26	61
Screening: age 45–79 y, 2 y	10.83	8.80	2.45	1548	166.7	151.1	7.46	55
Screening: age 50–69 y, 2 y; 70–74 y, 3 y	10.79	7.67	2.94	982	105.0	92.9	6.34	75
Screening: age 50–69 y, 2 y; 70–79 y, 3 y	10.86	8.60	2.76	1126	117.3	104.0	6.49	66
Screening: age 45–49 y, 1 y; 50–69 y, 2 y; 70–74 y, 3 y	10.72	8.64	2.66	1457	167.6	153.4	7.29	62
Screening: age 45–49 y, 1 y; 50–69 y, 2 y; 70–79 y, 3 y	10.79	8.76	2.49	1597	179.5	164.2	7.46	56
Screening: age 45–49 y, 1 y; 50–69 y, 2 y	10.70	7.79	2.77	1379	161.0	147.4	7.24	66
Screening: age 45–49 y, 1 y; 50–74 y, 2 y	10.74	8.71	2.60	1534	173.9	159.4	7.32	60
Screening: age 45–49 y, 1 y; 50–79 y, 2 y	10.82	8.83	2.41	1741	191.2	175.4	7.52	54

BC—breast cancer; DCIS—ductal carcinoma in situ; y—years, NNS—number needed to screen; Ref—reference strategy.

Compared to no screening, the lifetime risk of dying from detected breast cancer can be reduced on average from 4.27% to 3.05% (absolute reduction of 1.22%) with established biennial mammography at ages 50–69 years. The greatest benefit in terms of reduction in breast cancer deaths is achieved by screening with annual mammography for women at ages 45–49 and biennial interval at ages 50–79.

Similarly, the potential harm or burden associated with total numbers of mammograms, positive or false-positive mammograms increases with the intensity and duration of mammography screening (Table 6). The total numbers of mammograms ranged from 903 (biennial at ages 50–69 years) to 1741 (annual at ages 45–49, biennial at ages 50–79) per 100 women screened.

### 3.3. Benefit–Harm Balance

**Figure 2:**
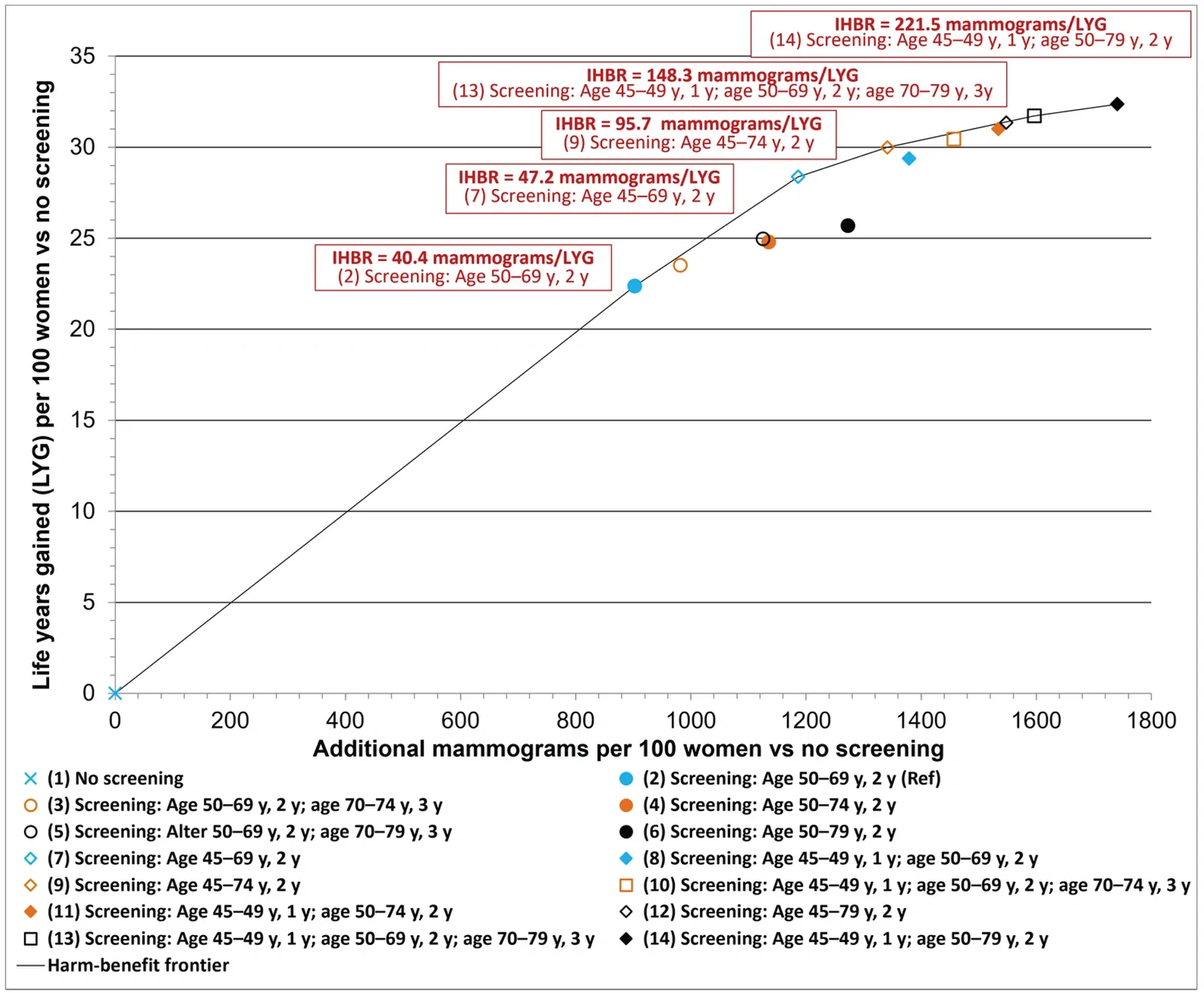
Harm–benefit trade-offs of evaluated mammography screening strategies for the German context: Efficiency frontier and incremental harm–benefit ratios measured in life years gained (LYG) vs. total number of additional mammograms per 100 women (based on [8]). IHBR—incremental harm–benefit ratio; LYG—life years gained; vs.—versus; pos.—positive; y—year.

Figure 2 shows the benefit–harm trade-offs of the evaluated screening strategies measured in additional mammograms associated with potential psychological and physical harms per life year gained (LYG). Compared to established screening with biennial mammography at ages 50–69, 47 additional mammograms per LYG must be accepted on the population level for lowering the age limit of mammography screening to 45 years (biennial screening at ages 45–69 years). Compared to this screening, additionally extending the upper age limit to 74 years (biennial screening at age 45–74 years) results in 96 additional mammograms per LYG. With a shorter screening interval at age 45–49 years and/or an extension of screening to age 79 years, only a comparatively small incremental benefit in terms of remaining life expectancy can be achieved with a substantial increase in the number of mammograms. The incremental harm–benefit ratios of these strategies are therefore significantly higher (Figure 2).

**Figure 3:**
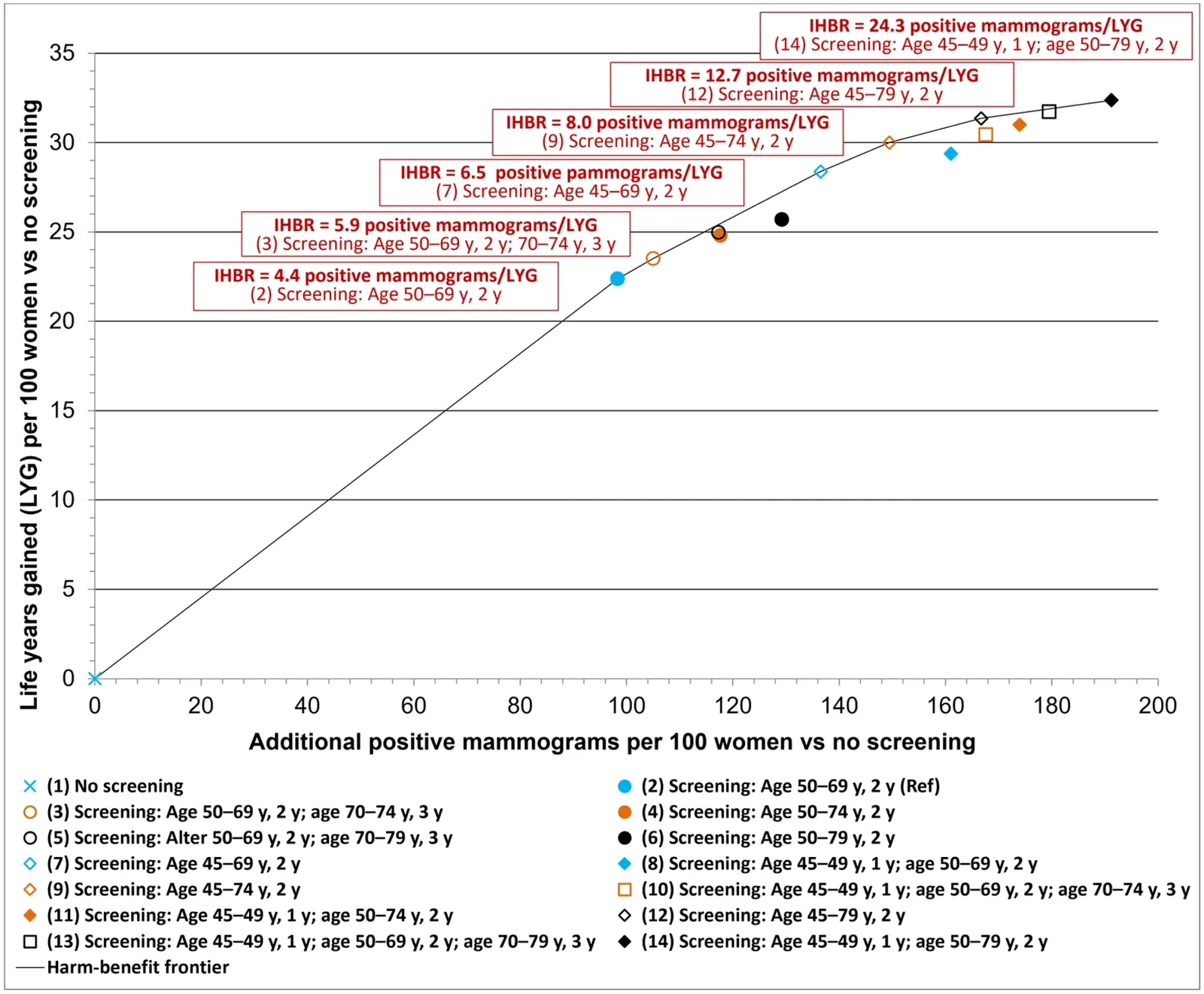
Harm–benefit trade-offs of evaluated mammography screening strategies for the German context: Efficiency frontier and incremental harm–benefit ratios measured in life years gained (LYG) vs. total number of additional positive mammograms per 100 women (based on [8]). IHBR—incremental harm–benefit ratio; BC—breast cancer; vs.—versus; pos.—positive; y—year.

**Figure 4:**
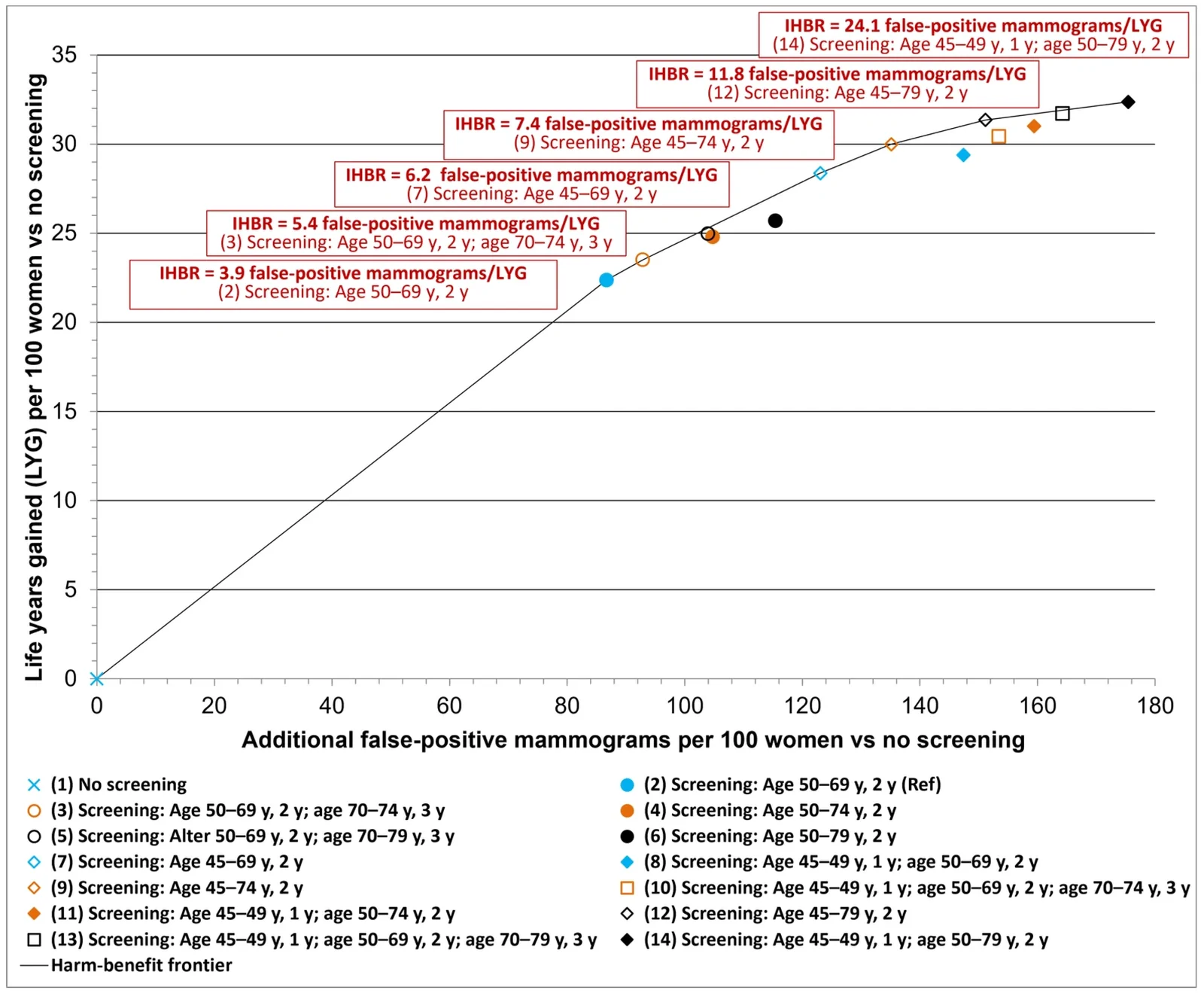
Harm–benefit trade-offs of evaluated mammography screening strategies for the German context: Efficiency frontier and incremental harm–benefit ratios measured in life years gained (LYG) vs. total number of additional false-positive mammograms per 100 women (based on [8]). IHBR—incremental harm–benefit ratio; BC—breast cancer; vs.—versus; pos.—positive; y—year.

Considering positive (Figure 3) or false-positive mammograms (Figure 4) as harm endpoints in relation to LYG, compared to established mammography screening (biennial, age 50–69 years), on average six positive mammograms or five false-positive mammograms per LYG must be accepted for an extension of screening to age 74 with a 3-year interval. Lowering the screening age to 45 years with a biennial interval results in around seven positive mammograms or six false-positive mammograms required to be accepted per LYG (Figure 3 and Figure 4).

Results of benefits in terms of prevented breast cancer deaths per 100 women relating to (a) number of additional mammograms per 100 women, (b) number of additional positive mammograms per 100 women and (c) number of false-positive mammograms per 100 women are presented in the Supplementary Materials (Figure S4).

### 3.4. Sensitivity Analyses

In sensitivity analyses, results were mostly robust when varying utility values across a wide range. Simultaneous variation in sensitivity and specificity in opposite directions in multi-way sensitivity analyses had a significant impact on the IHBRs. When specificity was reduced and sensitivity increased (scenario 1), the IHBRs increased. Whereas, when specificity was increased and sensitivity reduced, IHBRs decreased compared to the base-case analysis results, respectively (Supplementary Materials, Figure S5).

## 4. Discussion

This is the first model-based comparative effectiveness analysis within a benefit assessment of the IQWiG in Germany. We systematically evaluated evidence-based benefit–harm tradeoffs of different mammography screening strategies varying in the lower and upper age limits and in screening intervals.

We found that mammography screening at ages 45 to 79 years may prevent additional deaths from breast cancer and further increase remaining life expectancy. In this context, the earlier start of screening at 45 years of age has a greater benefit than the later end of screening at 79 years of age. Overall, both the benefits and potential harms increase with shorter screening intervals and extended screening duration within the strategies evaluated. Considering quality-adjusted life expectancy and explicit incremental benefit–harm ratios, biennial mammography screening in the age range of 45 to 74 years achieves a better benefit–harm balance compared to strategies with longer screening durations and shorter intervals.

Our results are consistent with recent findings of other studies [33,34] and the European Guidelines for breast cancer screening [5]. Results of a microsimulation model for the Canadian context show an earlier start of biennial screening at age 40 instead of age 50 to be cost saving [34]. The Collaborative Modelling Study of the USPSTF used a systematic review and six validated CISNET models to evaluate different breast cancer screening strategies [7,33]. Based on these results, the USPSTF currently recommends biennial screening mammography for women aged 40 to 74 years [5], with the model-based analyses suggesting that biennial mammography screening starting at age 40 years reduces breast cancer mortality by 30% compared with no screening [33]. The European Guidelines for breast cancer screening [5] currently recommend biennial or triennial mammography for women at age 45–49, biennial mammography for women at age 50–69, and triennial mammography for women at age 70–74. These recommendations are mainly based on evidence from randomized clinical trials with moderate certainty, quality-of-life data and false-positive findings based on observational studies with low and very low certainty.

A particular strength of this decision-analytic study is the transparent quantification of long-term trade-offs between health benefits and harms across different screening strategies that vary by starting and ending age of screening and screening intervals for specific age groups. For this purpose, we used both the quality-adjusted life expectancy approach and the incremental benefit–harm trade-off approach using selected explicit benefit and harm endpoints. As such, this modeling study provides an evidence-based, transparent and robust basis for health care decision-makers and complements results from randomized clinical trials, some of which may have heterogeneous study designs and conditions, and most of which do not track the effects of screening over a person’s lifetime.

Like all modeling studies, our evaluation has several limitations. Firstly, although several alternative mammography screening strategies were analyzed, not all possible alternative strategies were fully explored. Alternative screening strategies could alter the incremental harm–benefit ratio though and should therefore be considered in future studies [35]. Secondly, we did not include other imaging procedures such as ultrasound examinations or computer-based evaluations, tomosynthesis, or magnetic resonance imaging. Therefore, the conclusions drawn are based solely on the mammography screening strategies examined.

Thirdly, radiation-induced breast cancer development was not explicitly considered, and thus no conclusion can be made about its potential harm and its relation to benefit. However, the numbers of mammograms may serve as a proxy measure for radiation exposure. Fourth, despite basing the risk for breast cancer on age and breast density, familial or genetic risk for breast cancer or women’s menopausal status were not explicitly modeled. Consequently, the analysis cannot be applied to a population above average risk; for this purpose, separate modeling would be required.

Fifth, we used published international performance data stratified by age, breast density group and screening interval as well as German data for the distribution of the breast density group. However, sensitivity and specificity of mammography may vary across different countries and settings. Therefore, we varied mammography performance values across the range of reported uncertainty values in multiway-sensitivity analyses to assess the impact of uncertainty in mammography performance values on the benefit–harm ratios.

Most important, in Germany no empirical evidence exists on the acceptability threshold of how many harms for one additional unit of benefit screening-eligible women may consider appropriate. This acceptability threshold is likely to vary across women according to age, personal preferences, and perceptions of the burden of treatment-related consequences on quality of life. Acceptability thresholds of 150 overdiagnoses for a 10% reduction in breast cancer-specific mortality or 14 overdiagnoses or 47.8 false-positive screening findings for one additional breast cancer-related death prevented have been reported in the literature [36,37]. For women it is crucial that trade-offs are presented in shared decision-making and screening brochures. Also, women’s individual preferences and risk attitudes toward the acceptable incremental benefit–harm ratio should be considered and further research should establish a range for the harm–benefit acceptability thresholds of women in Germany for mammography screening.

The greatest uncertainty is expected in the results for overdiagnosis, as this is a measure for which there are currently no valid empirical data available [38,39]. Other models for the US context estimated that, depending on the screening strategy, 2–12% of breast cancer cases and 30–50% of DCIS cases could represent overdiagnosis [23]. Our model estimated the proportion of overdiagnosed cases (breast cancer and DCIS) to be 49–57%. Although absolute estimates for overdiagnosis from different models vary greatly across different studies, the results show that most overdiagnosed cases are DCIS. The model results for overdiagnosis are not directly comparable to other reported estimates from empirical studies as in the modeling study women are followed over their lifetime.

Importantly, based on the results of this modeling study, which complemented the benefit assessment conducted by the IQWiG [8], the G-BA in Germany declared in July 2024 that the upper age limit of mammography screening for the early detection of breast cancer is being extended [40]. Formerly women between the ages of 50 and 69 were eligible for attending mammography screening every two years. Since February 2025, women aged 70 to 75 also receive an invitation for biennial mammography screening [40]. An additional extension of the lower age limit for mammography screening is currently being discussed. According to the Federal Office for Radiation Protection, extending the age limits of mammography screening starting at age 45 and continuing until age 75 is justified from a radiation perspective, as the benefits outweigh the risks [41].

In future studies, the cost effectiveness of the different screening strategies should be assessed, including all relevant resource consumption aspects. For this, our decision-analytic model can be adapted considering all costs associated with screening, diagnostic follow-up of positive test results, and treatment procedures including (pre-)cancer treatment. In addition, decision-analytic studies should examine other risk-stratified screening strategies, including personalized strategies that take into account a woman’s screening history and other individual risk factors (genetics, menopausal status, other risk factors).

## 5. Conclusions

In conclusion, extending biennial mammography screening in Germany to women aged 45 to 74 years may prevent additional breast cancer deaths at an acceptable benefit–harm balance and improve quality-adjusted life expectancy. Future research is needed to survey the social and individual acceptance of overdiagnosis or potential harm per additional death prevented by breast cancer.

## Data Availability

All data generated or analyzed during this study are included in this published article.

## Supplementary Materials

The following supporting information can be downloaded at: https://www.mdpi.com/article/10.3390/cancers18172750/s1. In the Supplementary Materials further information on methods (e.g., model parametrization and calibration methods) and results eincluding model calibration, and further results for the base-case as well as sensitivity analyses are reported. 1. Methods: 1.1. Model Calibration; 1.2. Parametrization; Table S1: Relative survival probability for patients with detected breast cancer; Table S2: Age-specific proportion (in %) of breast density categories in the female population in Germany; Table S3: Relative risk of breast cancer depending on age and breast density category; 2. Results: 2.1 Model Calibration; Figure S1: Calibration results: age-specific annual incidence rate of symptomatically detected DCIS per 100,000 women; Figure S2: Calibration results: age-specific annual incidence rate of symptomatically detected breast cancer per 100,000 women; Figure S3: Calibration results: UICC stage distribution of detected breast cancer cases; 2.2. Additional Base-Case and Sensitivity Analyses Results; Figure S4: Base-Case Results: Harm-benefit efficiency frontier for mammography screening; Figure S5: Sensitivity analysis results. Harm-benefit efficiency frontier for mammography screening.

## Abbreviations

The following abbreviations are used in this manuscript:

BC	breast cancer
DCIS	ductal carcinoma in situ
G-BA	German Federal Joint Committee
IHBR	incremental harm–benefit ratio
IQWiG	Institute for Quality and Efficiency in Health Care
LY	life year
LYG	life year gained
NNS	number needed to screen
QALYs	quality-adjusted life years
QoL	quality of life
UICC	Union for International Cancer Control
USPSTF	US Preventive Services Task Force
