# Supplementary material for: Age Limits of Breast Cancer Screening with Mammography—A Decision-Analytic Benefit–Harm Evaluation to Inform Decision Making for the German Context
Source: Cancers (Basel). 2026 Aug 25;18(17):2750. doi: 10.3390/cancers18172750 (PMC13565422; doi:10.3390/cancers18172750)
Supplement: Supplementary file 1 [file cancers-18-02750-s001.zip › cancers-4346273-supplementary.pdf]

Supplementary Material

1. Methods

1.1.Model Calibration

The transition from 'healthy' to 'DCIS (undetected)' was calibrated using age-specific incidence of DCIS before the introduction of screening (data from 2003[1]). As start parameters, calculated values from age-specific DCIS incidences of the year 2019, i.e., after the introduction of breast cancer screening, were used.

The transition probabilities from 'healthy' as well as from 'DCIS' to 'undetected invasive breast cancer UICC I' were calibrated using the observed age-specific incidence of detected breast cancer cases and observed stage distribution of detected breast cancer cases in Germany before the introduction of screening based on cancer registry data.[2]

The probability of progression of undetected breast cancer cases from UICC I to UICC II, from UICC II to UICC III, and from UICC III to UICC IV was calibrated such that the model predicted the observed age-specific breast cancer incidence and stage-specific distribution in Germany.[3] The probabilities from an Austrian modeling study [4] were used as initial parameter values for the calibration. The transition probabilities from undetected to detected breast cancer in the 'no screening' strategy of the model is based on the stage-specific probabilities from an Austrian modeling study.[4] These initial parameter values were adjusted such that the detected cancer cases calculated by the model reflect the observed age-specific breast cancer incidences[2] and the stage distribution of detected cancer cases[3] in Germany before screening introduction.

1.2.Parametrization

Table S1. Relative survival probability for patients with detected breast cancer.

Year after initial diagnosis	Relative survival probability after initial diagnosis of breast cancer [5]			
	**UICC I***	**UICC II***	**UICC III***	**UICC IV***
	1	1	1	1
1st year	1.000	0.998	0.983	0.739
2nd year	1.000	0.990	0.960	0.783
3rd year	1.000	0.979	0.954	0.763
4th year	1.000	0.979	0.958	0.790
5th year	1.000	0.977	0.961	0.811
6th year	1.000	0.979	0.960	0.834
7th year	1.000	0.978	0.962	0.852
8th year	0.997	0.980	0.969	0.856
9th year	0.997	0.980	0.967	0.884
10th year	0.993	0.978	0.966	0.882
11th year	0.994	0.982	0.970	0.896
12th year	0.994	0.977	0.967	0.967
13th year	0.990	0.980	0.977	0.905
14th year	0.991	0.979	0.974	0.914
15th year	0.991	0.981	0.978	0.948

UICC - Union for International Cancer Control classification
*Data was transferred to UICC

Table S2. Age-specific proportion (in %) of breast density categories in the female population in Germany [6-8].

	breast density category			
**Age (years)**	**ACR A** (%)	**ACR B** (%)	**ACR C** (%)	**ACR D** (%)
**45-49§**	4.8	31.9	55.3	8.0
**50–54**	6.1	38.7	48.3	6.9
**55–59**	7.4	45.5	41.3	5.8
**60–64**	8.6	48.5	37.9	5.0
**65–69**	9.45	50.0	36.1	4.45
**70+**	10.0	50.85	35.0	4.15

ACR – American College of Radiology

ACR A: almost completely fatty (< 25% glandular).

ACR B: scattered fibroglandular densities (25-50% glandular)

ACR C: irregularly dense (51-75% glandular)

ACR D: extremely dense (> 75% glandular).

§ calculated from the absolute differences of the age category 50-54 vs. 55-59 in the respective ACR category.

Table S3. Relative risk of breast cancer depending on age and breast density category [6]

**Age (years)**	**Breast density category**			
	**ACR A**	**ACR B**	**ACR C**	**ACR D**
**40-49§**	0.376	0.783	1.212	1.573
**50–59**	0.388	0.807	1.251	1.623
**60–64**	0.400	0.832	1.291	1.675
**65–69**	0.581	0.885	1.228	1.283
**70+**	0.600	0.914	1.268	1.325

ACR –American College of Radiology

ACR A: almost completely fatty (< 25% glandular).

ACR B: scattered fibroglandular densities (25-50% glandular)

ACR C: irregularly dense (51-75% glandular)

ACR D: extremely dense (> 75% glandular).

§ calculated with relative weights of age categories (50-54y) to (55-59y): Factor 0.376 - 1.573

2. Results

2.1.Model Calibration

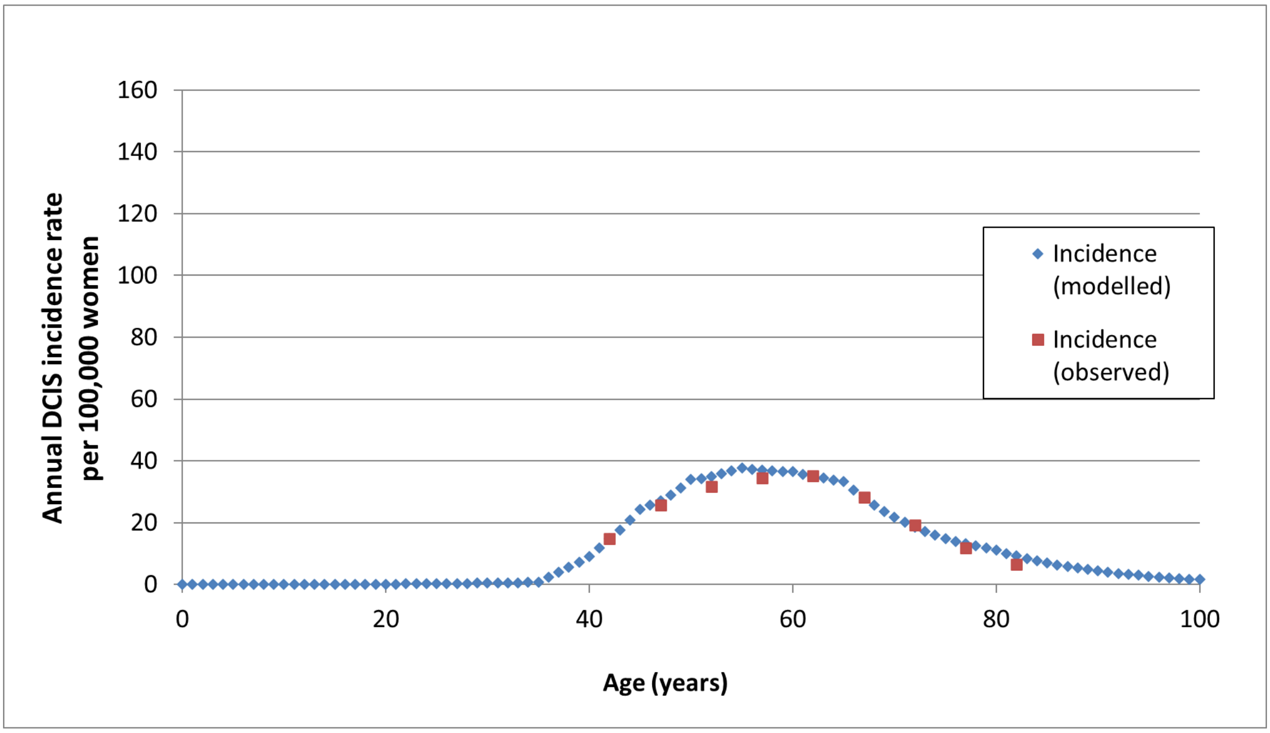

Figure S1. Calibration results: age-specific annual incidence rate of symptomatically detected DCIS per 100,000 women.

Shown are the annual new DCIS cases calculated by the decision-analytic model in the "no screening" strategy compared to observed annual new DCIS cases before the introduction of the breast cancer screening program in Germany from 1999-2004 [2] each per 100,000 women as a function of age. DCIS - Ductal Carcinoma in situ

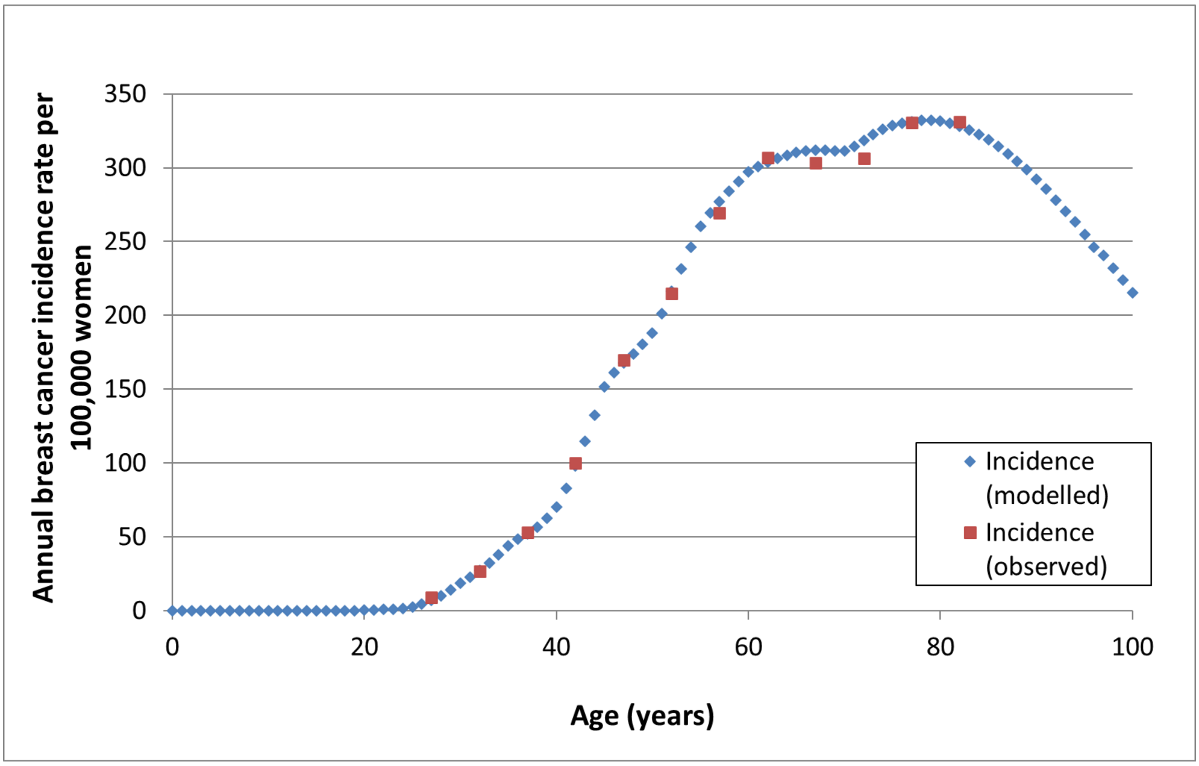

Figure S2. Calibration results: age-specific annual incidence rate of symptomatically detected breast cancer per 100,000 women.

Shown are new cancer cases calculated by the decision-analytic model in the "no screening" strategy compared with observed new cancer cases per year and 100,000 women before the introduction of the breast cancer screening program in Germany from 1999-2004[2], each per 100,000 women as a function of age.

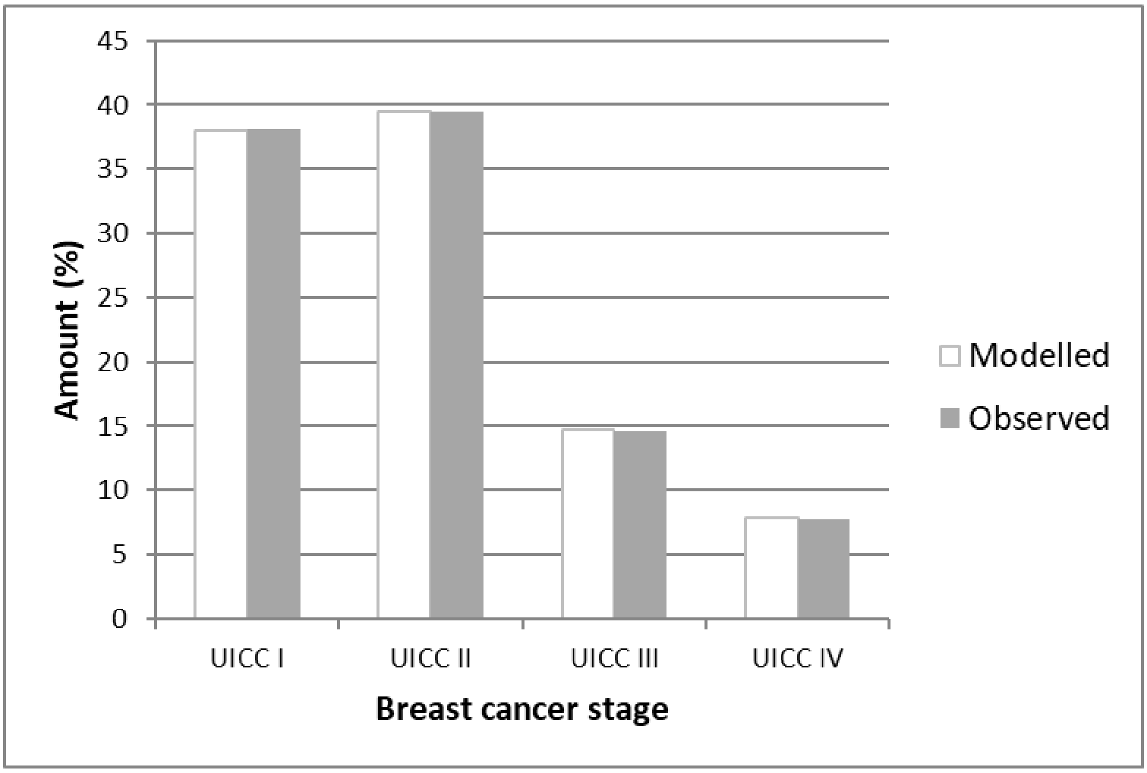

Figure S3. Calibration results: UICC stage distribution of detected breast cancer cases.

Shown is the proportion of women diagnosed with stage UICC I to IV breast cancer in the "no screening" strategy calculated by the decision analytic model compared to observed data before the introduction of the breast cancer screening program in Germany.[3]
UICC - Union for International Cancer Control classification

2.2. Additional Base-Case and Sensitivity Analyses Results

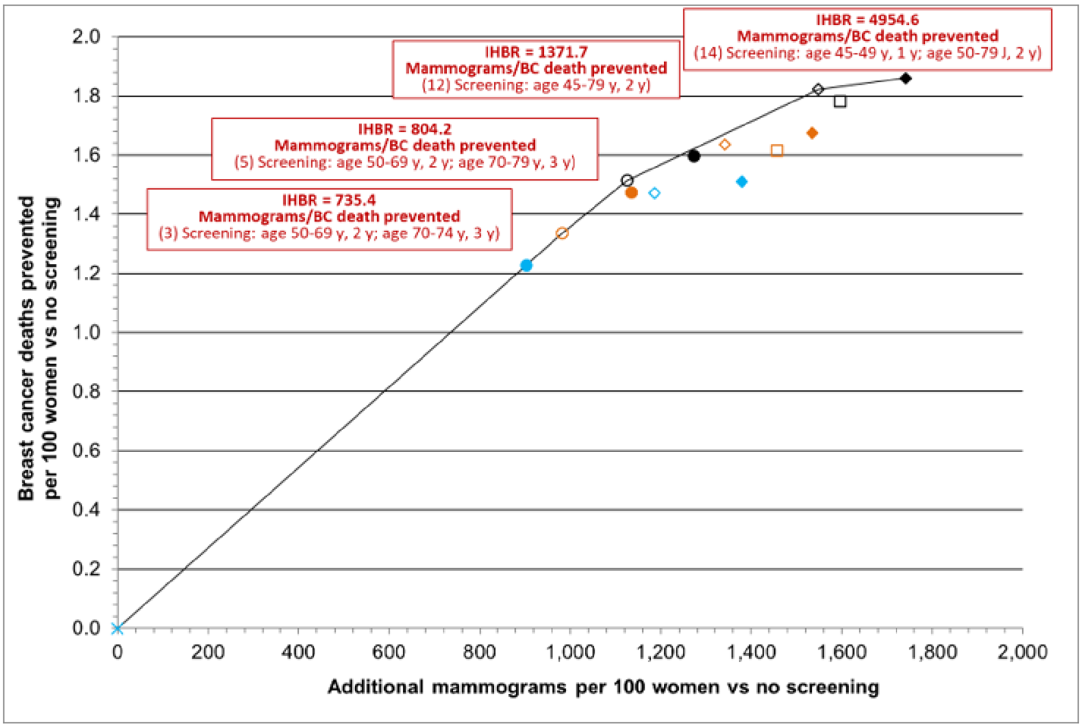
IHBR = 4954.6
Mammograms/BC death prevented
(14) Screening: age 45-49 y, 1 y; age 50-79 J, 2 y)
IHBR = 1371.7
Mammograms/BC death prevented
(12) Screening: age 45-79 y, 2 y)
IHBR = 804.2
Mammograms/BC death prevented
(5) Screening: age 50-69 y, 2 y; age 70-79 y, 3 y)
IHBR = 735.4
Mammograms/BC death prevented
(3) Screening: age 50-69 y, 2 y; age 70-74 y, 3 y)
Breast cancer deaths prevented per 100 women vs no screening
Additional mammograms per 100 women vs no screening
0.0
0.2
0.4
0.6
0.8
1.0
1.2
1.4
1.6
1.8
2.0
0
200
400
600
800
1,000
1,200
1,400
1,600
1,800
2,000

a)

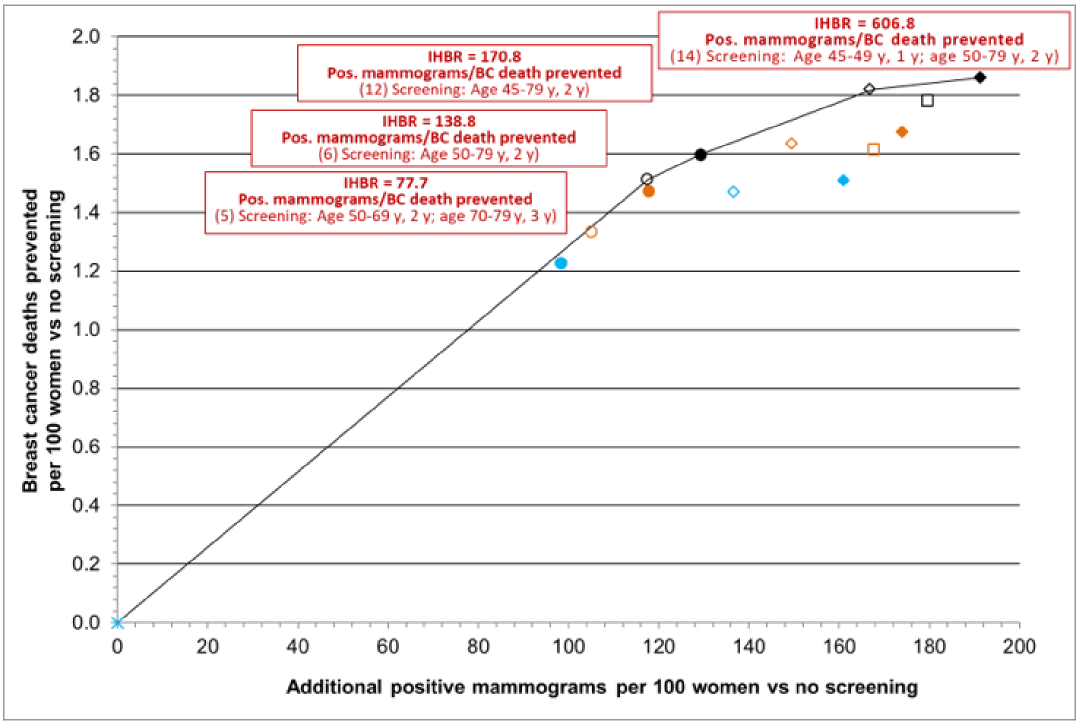
IHBR = 606.8
Pos. mammograms/BC death prevented
(14) Screening: Age 45-49 y, 1 y; age 50-79 y, 2 y)
IHBR = 170.8
Pos. mammograms/BC death prevented
(12) Screening: Age 45-79 y, 2 y)
IHBR = 138.8
Pos. mammograms/BC death prevented
(6) Screening: Age 50-79 y, 2 y)
IHBR = 77.7
Pos. mammograms/BC death prevented
(5) Screening: Age 50-69 y, 2 y; age 70-79 y, 3 y)
Breast cancer deaths prevented per 100 women vs no screening
Additional positive mammograms per 100 women vs no screening
0.0
0.2
0.4
0.6
0.8
1.0
1.2
1.4
1.6
1.8
2.0
0
20
40
60
80
100
120
140
160
180
200

b)

c)

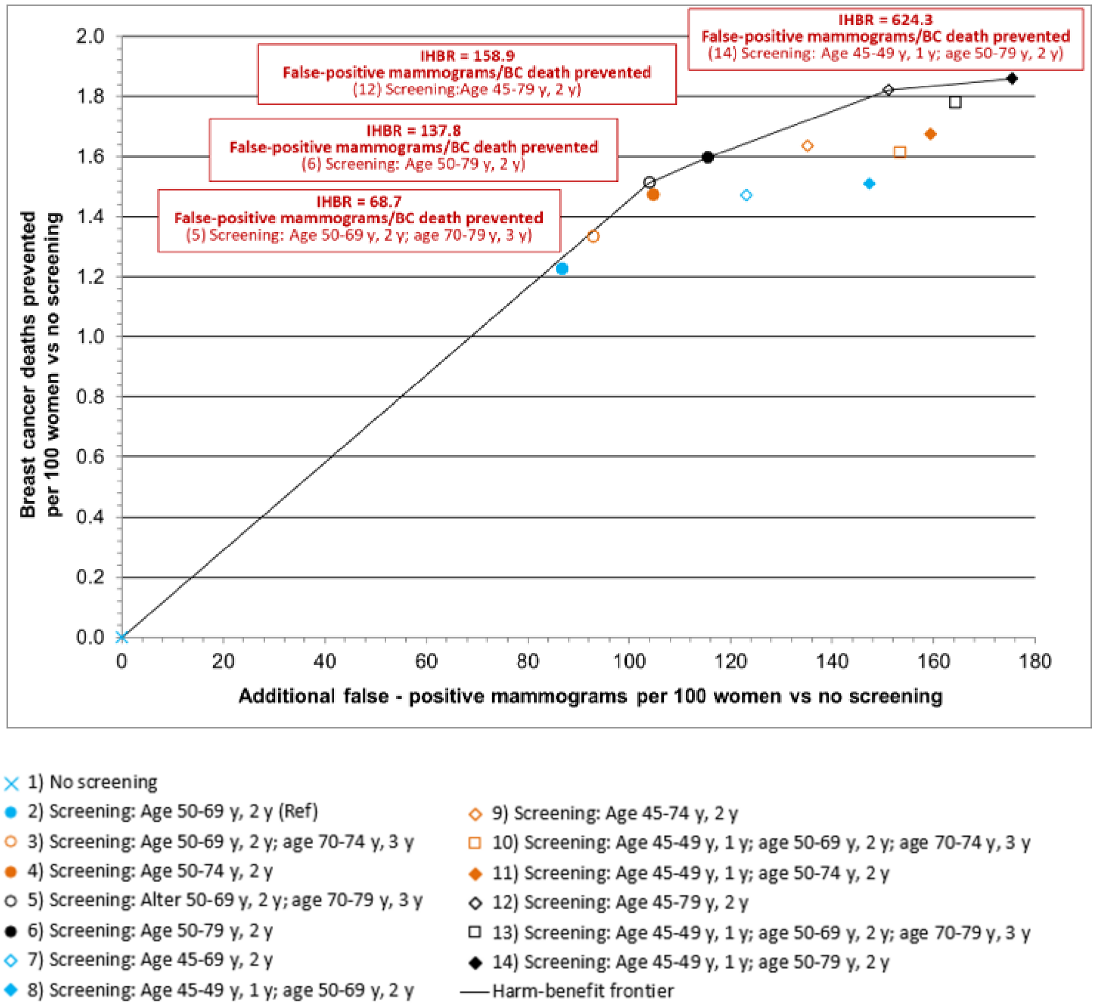

Figure S4. Base-Case Results: Harm-benefit efficiency frontier for mammography screening
Breast cancer deaths prevented per 100 women compared with a) number of additional mammograms per 100 women, b) number of additional positive mammograms per 100 women and c) number of additional false-positive mammograms per 100 women for different mammography screening strategies in women in Germany.
IHBR – incremental harm-benefit ratio; BC – breast cancer; vs – versus; pos. – positive; y – year

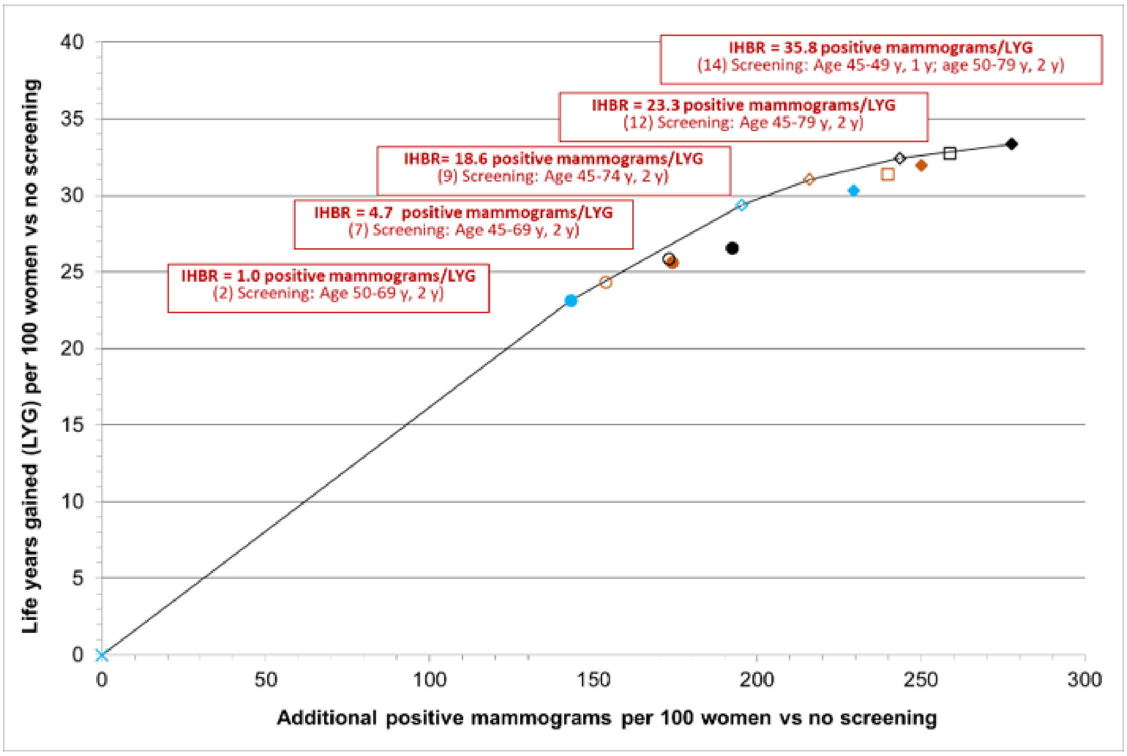
IHBR = 35.8 positive mammograms/LYG
(14) Screening: Age 45-49 y, 1 y; age 50-79 y, 2 y)
IHBR = 23.3 positive mammograms/LYG
(12) Screening: Age 45-79 y, 2 y)
IHBR= 18.6 positive mammograms/LYG
(9) Screening: Age 45-74 y, 2 y)
IHBR = 4.7 positive mammograms/LYG
(7) Screening: Age 45-69 y, 2 y)
IHBR = 1.0 positive mammograms/LYG
(2) Screening: Age 50-69 y, 2 y)
Life years gained (LYG) per 100 women vs no screening
Additional positive mammograms per 100 women vs no screening

a)

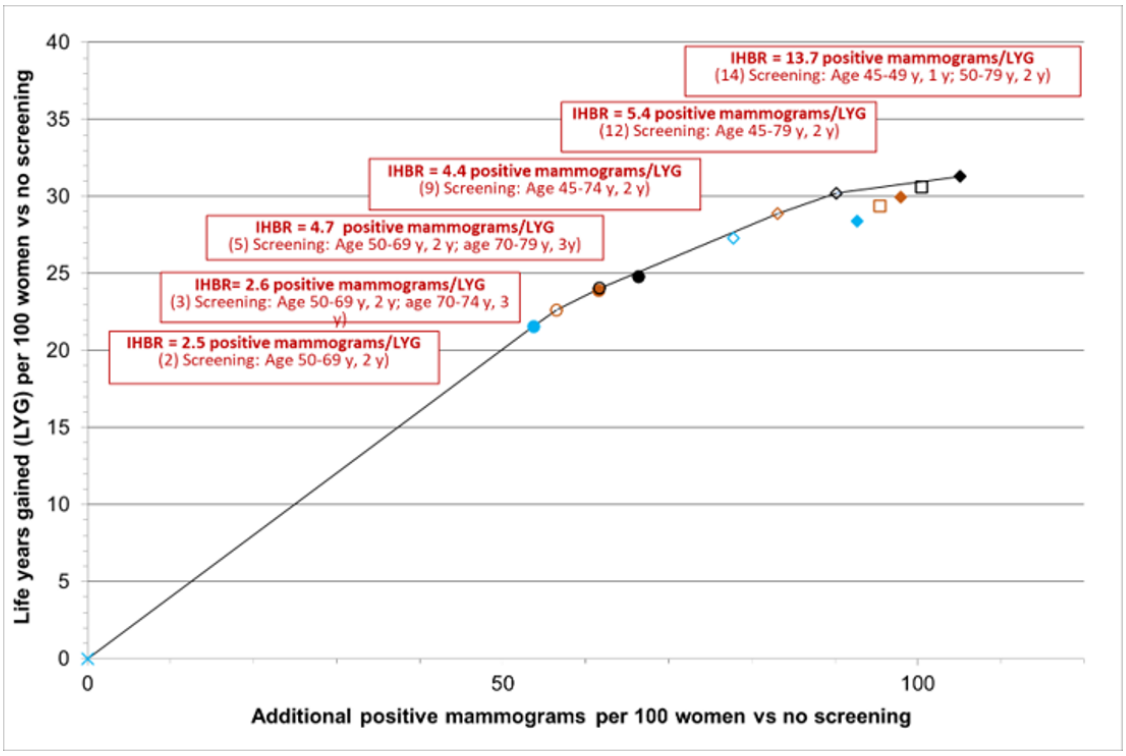
IHBR = 13.7 positive mammograms/LYG
(14) Screening: Age 45-49 y, 1 y; 50-79 y, 2 y)
IHBR = 5.4 positive mammograms/LYG
(12) Screening: Age 45-79 y, 2 y)
IHBR = 4.4 positive mammograms/LYG
(9) Screening: Age 45-74 y, 2 y)
IHBR = 4.7 positive mammograms/LYG
(5) Screening: Age 50-69 y, 2 y; age 70-79 y, 3y)
IHBR= 2.6 positive mammograms/LYG
(3) Screening: Age 50-69 y, 2 y; age 70-74 y, 3 y)
IHBR = 2.5 positive mammograms/LYG
(2) Screening: Age 50-69 y, 2 y)
Life years gained (LYG) per 100 women vs no screening
Additional positive mammograms per 100 women vs no screening

b)

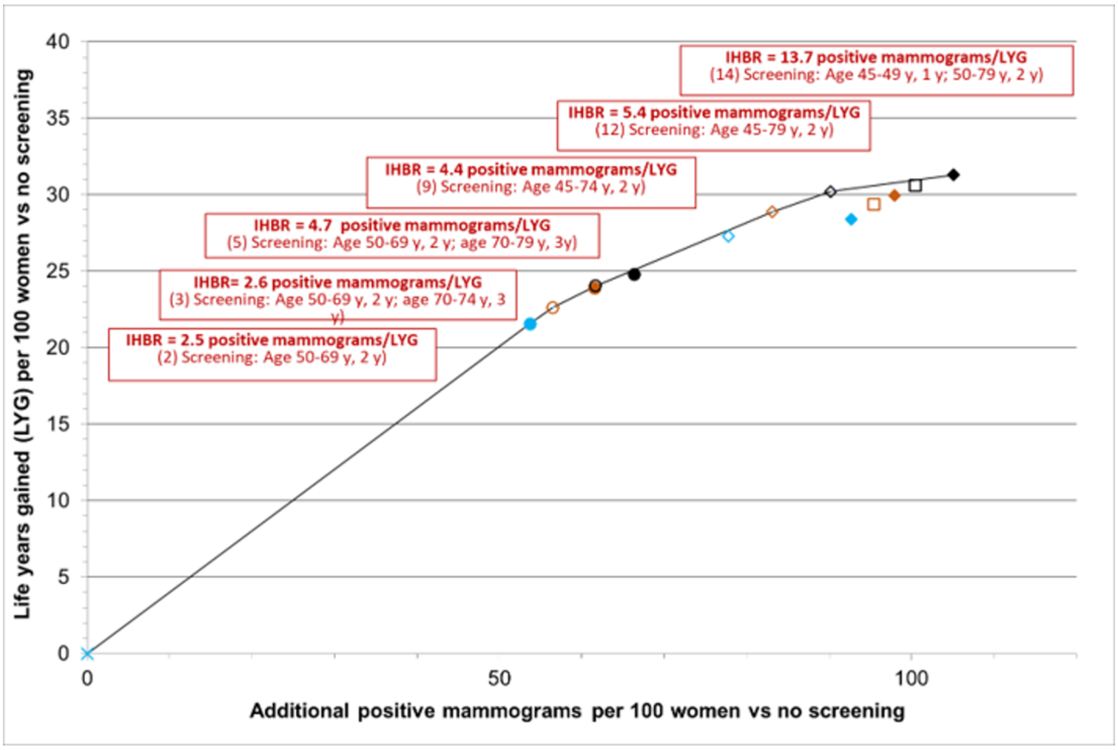

c)

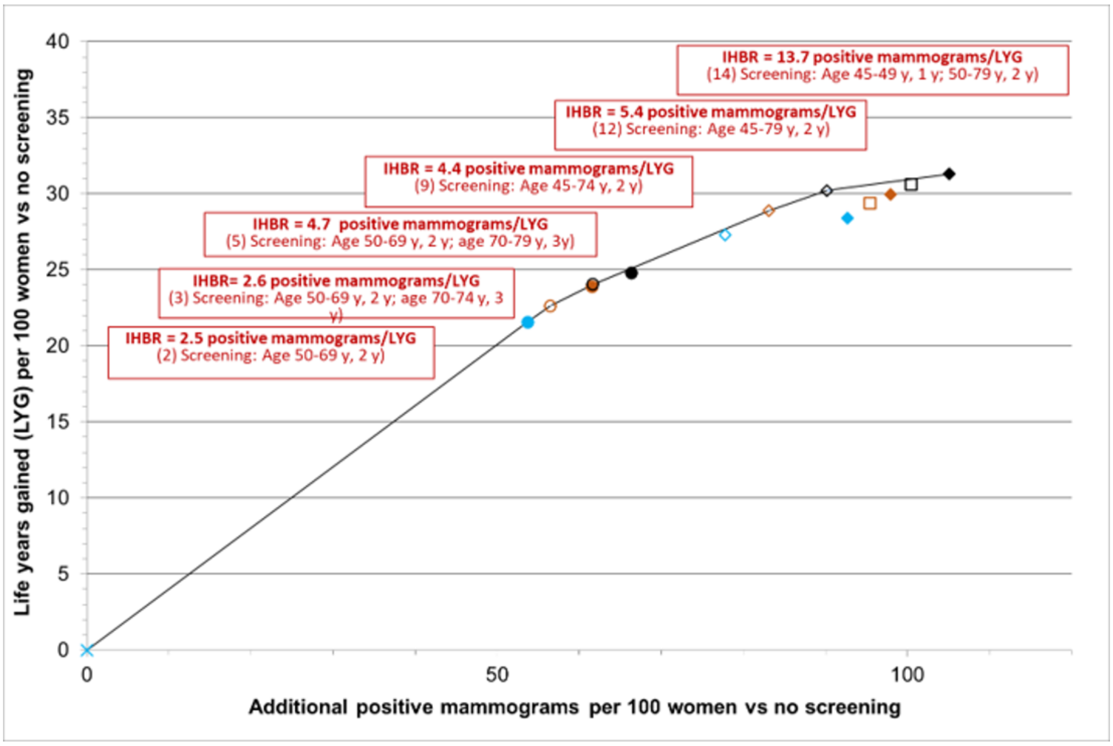

d)

1) No screening
2) Screening: Age 50-69 y, 2 y (Ref)
3) Screening: Age 50-69 y, 2 y; age 70-74 y, 3 y
4) Screening: Age 50-74 y, 2 y
5) Screening: Alter 50-69 y, 2 y; age 70-79 y, 3 y
6) Screening: Age 50-79 y, 2 y
7) Screening: Age 45-69 y, 2 y
8) Screening: Age 45-49 y, 1 y; age 50-69 y, 2 y
9) Screening: Age 45-74 y, 2 y
10) Screening: Age 45-49 y, 1 y; age 50-69 y, 2 y; age 70-74 y, 3 y
11) Screening: Age 45-49 y, 1 y; age 50-74 y, 2 y
12) Screening: Age 45-79 y, 2 y
13) Screening: Age 45-49 y, 1 y; age 50-69 y, 2 y; age 70-79 y, 3 y
14) Screening: Age 45-49 y, 1 y; age 50-79 y, 2 y
Harm-benefit frontier

Figure S5. Sensitivity analysis results. Harm-benefit efficiency frontier for mammography screening

Life years gained per 100 women compared with a) number of additional positive mammograms per 100 women in scenario 1 with a simultaneous increase in sensitivity and reduction in specificity by an absolute 5% b) number of additional positive mammograms per 100 women in scenario 2 a simultaneous decrease in sensitivity and increase in specificity by an absolute 5% c) number of false-positive mammograms per 100 women in scenario 1 with a simultaneous increase in sensitivity and reduction in specificity by an absolute 5% d) number of false-positive mammograms per 100 women in scenario 2 a simultaneous decrease in sensitivity and increase in specificity by an absolute 5%. IHBR – incremental harm-benefit ratio; BC – breast cancer; vs – versus; pos. – positive; y – year

References

1. Statistisches Bundesamt. Genesis-Online Datenbank. Krankenhauspatienten: Deutschland, Jahre, Geschlecht, Altersgruppen, Hauptdiagnose ICD-10 (1-3-Steller Hierarchie) und Tabelle Patienten in Vorsorge- oder Rehabilitationseinrichtungen: Deutschland, Jahre, Geschlecht, Altersgruppen, Hauptdiagnose ICD-10 (1-3-Steller Hierarchie). https://www-genesis.destatis.de/genesis/online. Accessed 5 July 2021.
2. Robert Koch Institut. Zentrum für Krebsregisterdaten: Datenbankabfrage mit Schätzung der Inzidenz, Prävalenz und des Überlebens von Krebs in Deutschland auf Basis der epidemiologischen Landeskrebsregisterdaten. Mortalitätsdaten bereitgestellt vom Statistischen Bundesamt. https://www.krebsdaten.de/Krebs/SiteGlobals/Forms/Datenbankabfrage/datenbankabfrage_stufe2_form.html. Accessed 24 June 2021.
3. Epidemiologisches Krebsregister NRW. Schwerpunktbericht 2. Brustkrebs - Ergebnisse aus dem Reg.-Bez. Münster 1992-2004. https://www.landeskrebsregister.nrw/fileadmin/user_upload/dokumente/veroeffentlichungen/ekr_nrw_Brustkrebs_web.pdf; Accessed 20 September 2021.: Epidemiologisches Krebsregister NRW.
4. Schiller-Fruehwirth I, Jahn B, Einzinger P, et al. The Long-Term Effectiveness and Cost Effectiveness of Organized versus Opportunistic Screening for Breast Cancer in Austria. Value Health. 2017;20(8):1048-1057.
5. Tumorregister München. Überleben ICD-10 C50: Mammakarzinom (Frauen) [Internet]. https://www.tumorregister-muenchen.de/facts/surv/sC50f_G-ICD-10-C50-Mammakarzinom-Frauen-Survival.pdf. Accessed 17 January 2022.
6. Arnold M, Pfeifer K, Quante AS. Is risk-stratified breast cancer screening economically efficient in Germany? PLOS ONE. 2019;14(5):e0217213.
7. Weigel S, Heindel W, Heidrich J, et al. Digital mammography screening: sensitivity of the programme dependent on breast density. Eur Radiol. 2017;27(7):2744-2751.
8. Kerlikowske K, Ichikawa L, Miglioretti DL, et al. Longitudinal measurement of clinical mammographic breast density to improve estimation of breast cancer risk. J Natl Cancer Inst. 2007;99(5):386-395.
